# MRI-based radiomic clustering identifies a glioblastoma subtype enriched for neural stemness and proliferative programs

**DOI:** 10.3389/fonc.2025.1662401

**Published:** 2025-11-25

**Authors:** Zhongyi Zhang, Yang Liu, Zhicong Zhang, Tian Gui, Youhui Chen, Qian Chen, Xiufu Wu, Li Sun, Sujie Li, Shuyang Wei

**Affiliations:** 1 Department of Neurosurgery, The Second Affiliated Hospital of Guilin Medical University, Guilin, China; 2 Department of Operating Theatre, The Second Affiliated Hospital of Guilin Medical University, Guilin, China; 3 Department of Neurosurgery, The First Affiliated Hospital of Guilin Medical University, Guilin, China

**Keywords:** radiogenomics, glioblastoma, single-cell transcriptomics, spatial transcriptomics, neural stem-like cells, tumor heterogeneity

## Abstract

**Introduction:**

Glioblastoma (GBM) is a highly aggressive brain tumor with a median survival of only 15 months. A major challenge in GBM management is the pronounced inter- and intratumoral heterogeneity, which complicates prognosis and therapy. Radiomics, the quantitative extraction of features from medical images, can capture this heterogeneity across the entire tumor volume, but the biological basis of radiographic phenotypes remains poorly understood.

**Methods:**

We integrated preoperative MRI-based radiomic stratification with multi-platform transcriptomics (bulk RNA-seq, single-cell RNA-seq, and spatial transcriptomics) in IDH-wildtype GBM patients. Unsupervised clustering of radiomic features identified four imaging subtypes.

**Results:**

Group 4 emerged as a high-risk subtype associated with significantly worse survival and a distinctive MRI pattern of peripheral contrast enhancement. Transcriptomic analyses revealed that Group 4 tumors were enriched in cell-cycle and proliferation markers and exhibited neural stem cell–like gene expression signatures. Single-cell profiling confirmed an elevated proportion of stem-like malignant cells in this subtype. Spatial transcriptomics further demonstrated that these proliferative, stem-like programs were localized predominantly to the tumor periphery, corresponding to the rim-enhancing regions on MRI. Finally, we identified the developmental transcription factor VAX2 as a candidate driver of the Group 4 gene network; functional assays showed that VAX2 promotes GBM cell proliferation in vitro.

**Discussion:**

Our findings link a radiomics-defined MRI phenotype to specific molecular programs and cell populations in GBM, suggesting that radiomic subtypes can serve as noninvasive biomarkers of tumor biology and highlighting potential therapeutic targets in aggressive, stem-like tumor cell populations.

## 1 Introduction

Glioblastoma (GBM) is the most lethal primary brain tumor, with a median survival of 12–15 months despite standard therapy (1). One major factor underlying this poor prognosis is the extreme inter- and intra-tumor molecular heterogeneity of GBM (2). GBM tumors contain diverse cellular subpopulations; for example, a small fraction of glioma stem-like cells is thought to drive therapy resistance and recurrence (3). Furthermore, GBM is characterized by diffuse infiltration of malignant cells into surrounding brain tissue (4). Most tumors recur locally, often arising from residual cells at the resection margin (3). This biological complexity poses a significant challenge for clinical management, as single-sample assays may miss the most aggressive populations.

Magnetic resonance imaging (MRI) is a standard tool for GBM diagnosis and monitoring, providing a whole-tumor view (5). However, conventional radiological interpretations offer limited insight into tumor biology and can be subjective (6). Radiomics has emerged as an approach to objectively quantify imaging features, converting images into high-dimensional data that capture tumor morphology and texture (7). In GBM, radiomic features have shown promise for prognostication – for example, radiomic risk scores can stratify patients by survival risk in both discovery and validation cohorts (8). Yet, the radiogenomic associations (linking imaging phenotypes to molecular profiles) remain largely unexplored and inconsistent (6). Recent studies suggest that imaging subtypes defined by radiomics may reflect distinct genomic programs (2). This implies that quantitative MRI phenotypes could serve as surrogates for underlying tumor biology, but a deeper multi-scale annotation of radiographic features is needed to fully realize this potential.

Integrating radiomic data with modern transcriptomic profiling offers an opportunity to better interpret imaging phenotypes in biological terms. Bulk RNA sequencing provides an overview of gene expression in tumor tissue, whereas single-cell RNA sequencing (scRNA-seq) can resolve the diverse cell types and states within a tumor (4). Notably, scRNA-seq studies have identified multiple coexisting malignant cell states in GBM, including neural-progenitor-like, oligodendrocyte progenitor–like, astrocyte-like, and mesenchymal-like cells, which can each contribute to tumor growth (1). Spatial transcriptomics adds another dimension by mapping gene expression to specific locations in the tumor, revealing how molecular programs differ between the hypoxic tumor core and the invasive margin (4). Despite these advances, few studies integrate radiomics with single-cell or spatial data, and relationships to spatially resolved cellular architecture remain unclear.

Here, we address this gap by investigating how radiomics-defined MRI phenotypes correlate with underlying gene expression programs at bulk, single-cell, and spatial levels in GBM. We performed unsupervised clustering of radiomic features extracted from preoperative MRIs to define imaging-based GBM subtypes. We then integrated these imaging subtypes with transcriptomic profiles, including bulk tumor RNA-seq as well as scRNA-seq and spatial transcriptomics from representative tumors, to characterize the molecular and cellular features associated with each radiomic class. In particular, we focused on a radiomics-defined high-risk subgroup and identified candidate transcriptional regulators of its aggressive phenotype. Finally, we conducted functional experiments to validate the role of a novel transcription factor (VAX2) implicated by our analysis. By combining noninvasive imaging with multi-omics, this study aims to clarify GBM heterogeneity and identify imaging-linked biomarkers for prognosis and therapy.

## 2 Materials and methods

### 2.1 MRI image acquisition and patient selection

A total of 61 preoperative MRI scans of GBM patients with complete clinical annotations were obtained from The Cancer Imaging Archive (TCIA) (9). Patient demographics and clinical features, including age, sex, overall survival (OS), and progression-free survival (PFS).

### 2.2 Tumor segmentation and radiomic feature extraction

MRI images were segmented using 3D Slicer software (version 4.11) (10), manually delineating four tumor subregions: contrast-enhancing tumor (ET), non-enhancing tumor (NET), necrotic core (NEC), and edema (ED). Segmentations were performed independently by two neurosurgeons and adjudicated by a senior neurosurgeon; discrepancies were resolved by consensus, and the consensus masks were used for all analyses. While formal Dice/ICC were not computed, this procedure ensured consistent ROI definitions. Radiomic features were extracted using PyRadiomics (version 3.0.1) (11), yielding 106 features. Features with zero variance were excluded; missing values were imputed by the feature-wise median, and the feature matrix was standardized by column-wise z-scoring. A comprehensive description of all radiomics preprocessing, segmentation, and feature extraction parameters is provided in **Supplementary Data Sheet 1** – Radiomics Workflow Description.

### 2.3 Radiomic clustering and prognostic analysis

Principal Component Analysis (PCA) (12) was first used to explore structure in the radiomic feature space. The optimal number of groups (n=4) was determined using the elbow method based on within-group sum of squares. To assess robustness beyond PCA, we performed consensus clustering using k-means with 100–150 resamples, sub-sampling 90% of subjects and 80% of features per iteration; the resulting consensus matrix supported a four-cluster solution. Prognostic significance was evaluated using Cox proportional hazards regression adjusting for age and sex. In addition to the primary Cox models, we conducted small-sample–robust sensitivity analyses: (i) Firth-penalized Cox for the prespecified contrast; (ii) restricted mean survival time (RMST) with τ determined from the follow-up distribution (τ=761 days); and (iii) proportional hazards diagnostics using Schoenfeld residuals.

### 2.4 Bulk RNA-seq data processing

Publicly available matched bulk RNA-seq data corresponding to the MRI-derived radiomic groups were analyzed. Differential expression analysis was performed using the Limma package (13), with significance defined as |log_2_ fold change| ≥ 1 and FDR < 0.05 (Benjamini–Hochberg adjusted). Functional annotation of DEGs was conducted using Gene Ontology (GO) (14) enrichment and GSEA (MSigDB Hallmark sets) (15).

### 2.5 Single-cell RNA-seq data analysis

Publicly available single-cell RNA-seq datasets from glioblastoma (GSE103224) (16) were processed using the Seurat package (version 5.0) (17). Clustering resolution was optimized using Clustree (18), selecting a resolution of 0.6 to define 22 transcriptionally distinct clusters. Cell-type annotation was performed using canonical markers and the scMayoMap (19). Pearson correlation analysis assessed the association between Group 4 specific DEGs and annotated cell-type marker genes.

### 2.6 Pseudotime trajectory analysis

Trajectory inference was conducted using Slingshot (20), with neural stem cells (NSCs) designated as the starting cell population based on correlation analysis. Lineage trajectories and pseudotime distributions were visualized to investigate cell developmental dynamics.

### 2.7 Spatial transcriptomics and deconvolution analysis

Spatial transcriptomic data were obtained from publicly available glioblastoma samples (GSE194329) (21) and processed using Seurat and SPATA2 (22) for cell-type deconvolution. Group 4 DEG module scores were computed and spatially visualized. Hotspot analysis and Moran’s I were utilized to assess spatial autocorrelation and clustering significance. The spatial transcriptomic maps were interpreted at a regional level rather than through voxel-wise registration with MRI. Specifically, spatial enrichment of Group 4 gene modules was evaluated relative to histologically defined tumor compartments (core *vs*. periphery) that correspond to the macroanatomical regions visible on MRI, acknowledging the difference in physical resolution between transcriptomic spots (55–100 µm) and MRI voxels (>1 mm³).

### 2.8 Cell culture and siRNA transfection

Human LN229 glioblastoma cells (ATCC, CRL-2611) were cultured according to supplier guidelines in Dulbecco’s modified Eagle medium (DMEM) supplemented with 10% FBS and antibiotics (penicillin/streptomycin). Cells were transfected with siRNAs targeting VAX2 using Lipofectamine RNAiMAX (Thermo Fisher) according to the manufacturer’s instructions. The siRNA sequences were: si-VAX2.1: 5’-UUCGGGAAAUUGUCCUGCC-3’, si-VAX2.2: 5’-GCAGAAGAAAGACCAGAGC-3’ (23).

A non-targeting scrambled siRNA (siNC) (SMARTpool) was used as control. Transfection efficiency and knockdown were validated by quantitative RT-PCR at 48 hours post-transfection.

### 2.9 Quantitative real-time PCR

Total RNA was extracted from LN229 cells using the TRIzol extraction Kit (Invitrogen) following the manufacturer’s instructions. cDNA synthesis was conducted with the Reverse Transcription Kit (Takara). Real-time PCR was performed using SYBR Green PCR Master Mix (Takara) on a QuantStudio 6 Real-Time PCR System (Thermo Fisher) under the following conditions: initial denaturation at 95°C for 5 min, followed by 40 cycles of 95°C for 30 sec, 60°C for 40 sec, and 72°C for 1 min.

VAX2 F: CAAGCGGACACGTACATCCTT, R: GCCGCAGAATGTTGGAGGT.

OTP F: CAGGCTAGGTATGAAAGATGCC, R: GAAGCAGGGGTAGAGCCCA.

C1QL2 F: CACCTGCCGCATGATCTGT, R: TGGTCCCTGGATAAACGGAGG.

GAPDH F: AGGTCGGTGTGAACGGATTTG, R: TGTAGACCATGTAGTTGAGGTCA was used as an internal reference gene.

### 2.10 Cell proliferation assay

Cell viability was assessed using the CellTiter-Glo^®^ Luminescent Cell Viability Assay kit (Promega), adhering closely to the manufacturer’s guidelines. LN229 cells were seeded in 96-well plates at 5×10³ cells per well and transfected as described above. Luminescence was measured at 24, 48, and 72 hours post-transfection using the POLARstar Optima Microplate Reader (BMG). Results were expressed as mean ± standard deviation from three independent replicates. Statistical significance was evaluated using one-way ANOVA followed by Tukey’s *post-hoc* tests.

## 3 Result

### 3.1 Radiomics-based grouping of GBM MRI features reveals distinct prognostic subgroups

**Figure 1:**
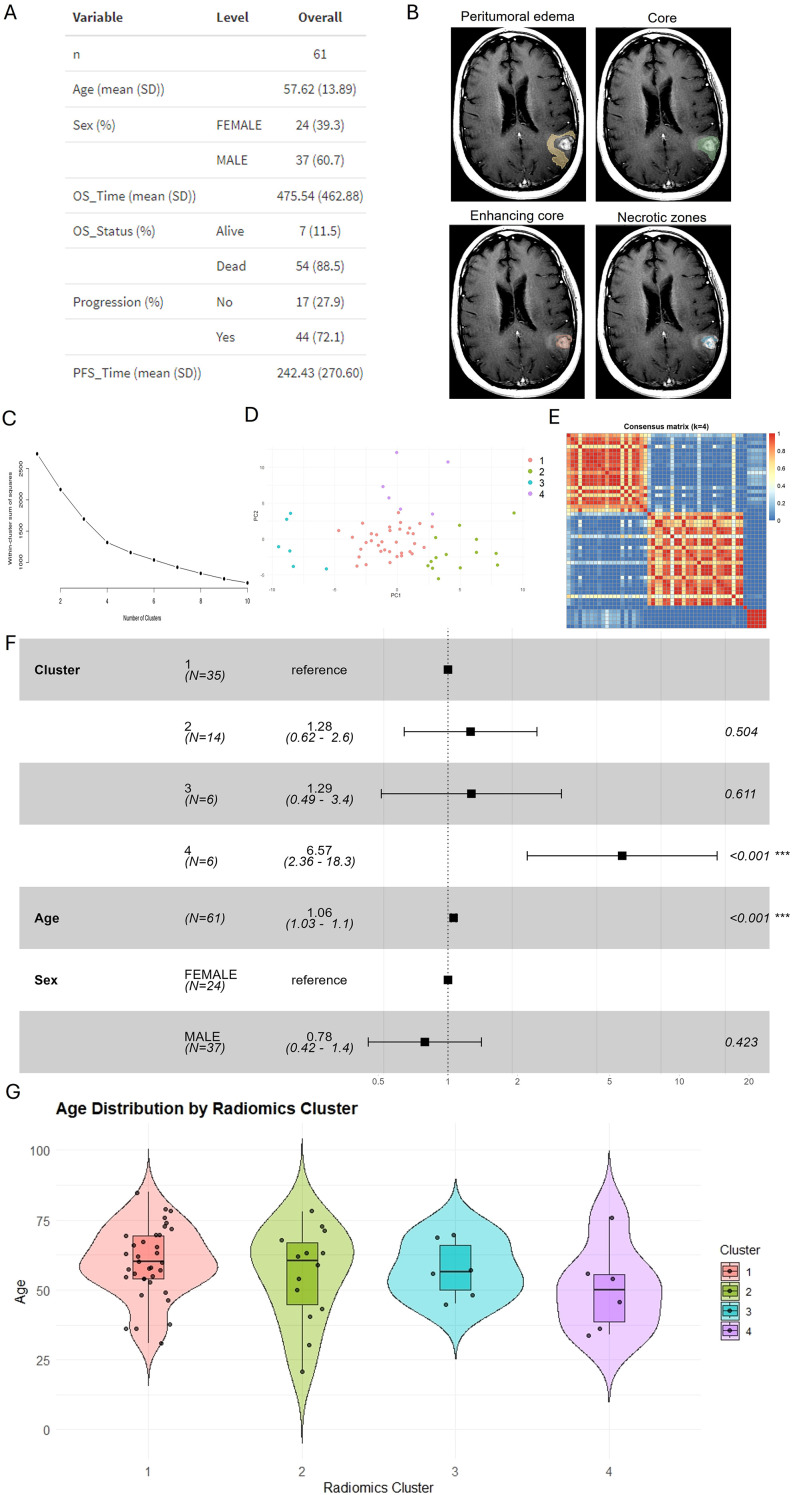
Radiomics-based clustering reveals a high-risk GBM subgroup with distinct prognosis. **(A)** Summary of clinical and demographic characteristics of the 61 GBM patients included in this study. n = 61 patients; descriptive statistics reported as mean (SD) unless noted. **(B)** Representative examples of manual MRI segmentation showing four distinct tumor subregions: ED, NET, ET, and NEC, annotated on T1-weighted post-contrast images. Segmentations by two neurosurgeons with senior adjudication; consensus masks used for all analyses. **(C)** Elbow plot showing within-cluster sum of squares for radiomic feature clustering, suggesting an optimal number of clusters at four. Elbow based on within-cluster sum of squares on z-scored features. **(D)** Principal component analysis (PCA) plot of radiomic features colored by cluster assignments (Cluster 1–4), demonstrating partial separation among radiomic subtypes. **(E)** Consensus clustering of radiomic features (k = 4; 100–150 resamples; 90% subjects × 80% features per run; k-means; Euclidean). The consensus matrix shows four well-defined within-cluster blocks with high mean co-assignment probabilities (average within-cluster consensus > 0.8), supporting the robustness of the four-group structure. PCA performed on z-scored features; points represent individual patients (n = 61). **(F)** Overall survival for radiomic groups, primary multivariable Cox analysis in the full cohort (n=61, adjusted for age and sex). Statistical estimates for complementary sensitivity analyses (complete-case, Firth, KM, RMST) are reported in the Results. Consensus clustering (k = 4; k-means; Euclidean; 100–150 resamples; 90% subjects × 80% features per run). **(G)** Age distribution and adjusted comparisons; the risk trend appears largely independent of age in sensitivity analyses, with precision limited by the small size of Group 4.

To investigate the prognostic relevance of MRI-derived radiomic features in GBM, we analyzed preoperative MRI scans from 61 patients retrieved from TCIA, all with complete clinical annotations. Clinical characteristics of the cohort are summarized in **Figure 1A**, with a mean age of 57.6 years (SD 13.9), male predominance (60.7%), and a high mortality rate (88.5%) at last follow-up. The median OS and PFS were approximately 476 and 242 days, respectively. Tumor subregions, including ED, ET, NET, and NEC, were manually segmented on MRI using 3D Slicer (**Figure 1B**).

A total of 106 radiomic features were extracted using the PyRadiomics package. After removing features with zero variance, missing values were median-imputed, and features were standardized by column-wise z-scoring. Unsupervised grouping was first performed using PCA, and an elbow plot of within-group sum of squares suggested an optimal cluster number of four (**Figure 1C**). PCA visualization showed a distinct distribution of patients across the four subtypes (**Figure 1D**). To assess robustness beyond PCA and elbow, consensus clustering (k-means; 100–150 resamples; 90% subjects × 80% features) produced a well-structured consensus matrix with four clear co-assignment blocks (**Figure 1E**). Bootstrap resampling yielded consistently high within-cluster consensus values (>0.8), supporting the robustness of the four-group structure despite the limited cohort size. (**Figure 1E**).

To determine the prognostic significance of these radiomic groups, we performed multivariable Cox proportional hazards analysis incorporating group assignment, age, and sex. Compared to Group 1, Group 4 was significantly associated with worse overall survival (HR = 6.57, 95% CI: 2.36–18.3; *p* < 0.001), indicating a high-risk phenotype (**Figure 1F**). To address precision concerns raised by the small size of Group 4, we performed complete-case sensitivity analyses with small-sample–robust estimators: Cox HR = 1.77 (95% CI 0.68–4.57; *p* = 0.239), Firth-penalized HR = 1.92 (95% CI 0.76–4.83; *p* ≈ 0.194), Kaplan–Meier log-rank p = 0.32, and RMST at τ = 761 days = −89 days for Group 4 *vs* Group 1 (95% CI −229 to 50; *p* = 0.209). The effect direction was consistent across analyses, with wider intervals expected under reduced sample size. Increasing age was independently associated with worse survival (HR = 1.06 per year; *p* < 0.001), consistent with known clinical patterns in GBM, whereas sex was not significantly associated with survival outcomes.

In the primary adjusted model (age and sex), the association between Group 4 and worse overall survival persisted. Given the small size of Group 4, estimates remain imprecise, and residual confounding by unmeasured factors (e.g., extent of resection, MGMT status, treatment regimen) cannot be excluded. Accordingly, we refrain from asserting age-independence and interpret these results cautiously (**Figure 1G**).

### 3.2 Radiomic features underlying the high-risk Group 4 subtype

**Figure 2:**
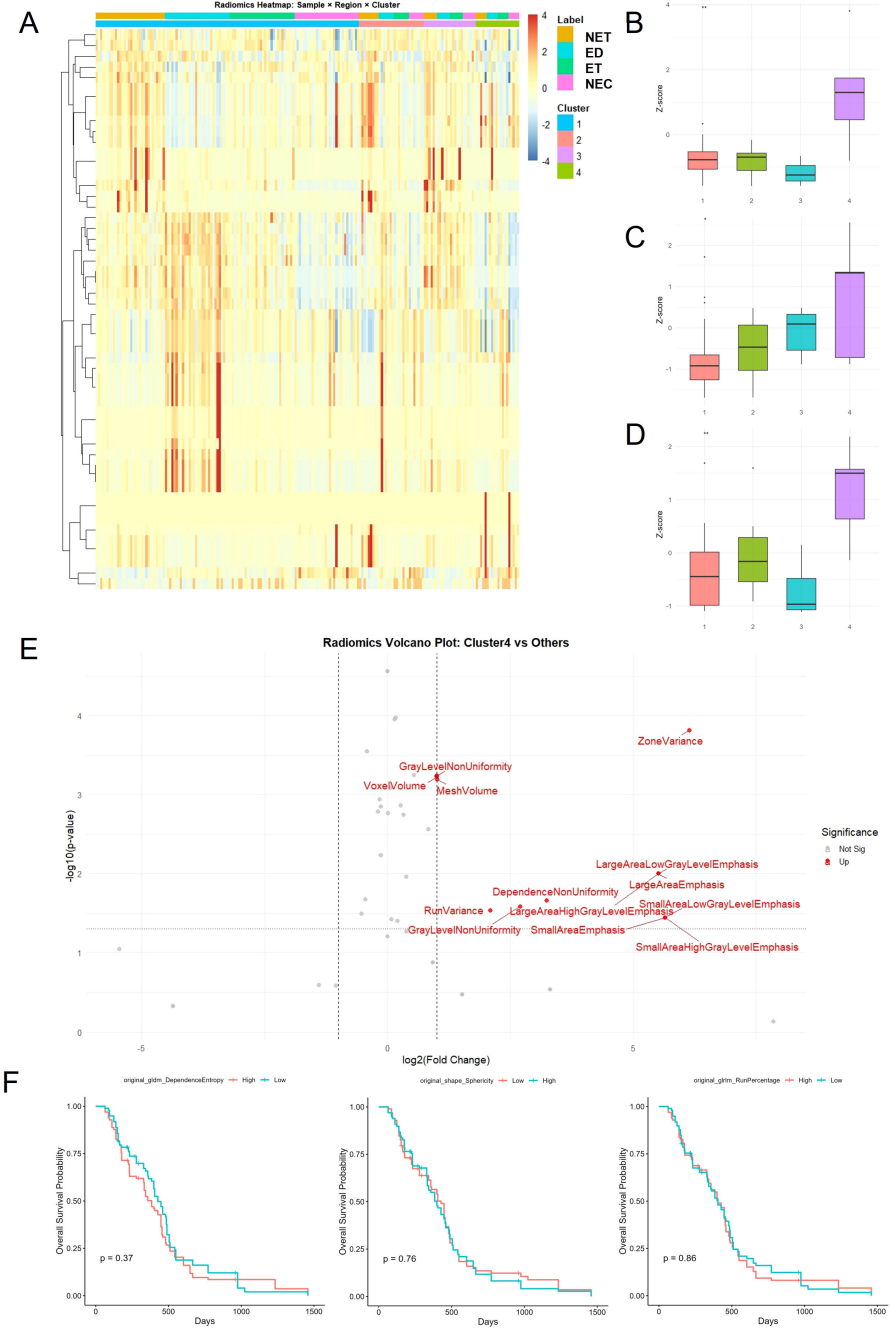
Radiomic feature profiling reveals distinctive patterns in high-risk Cluster 4. **(A)** Heatmap of z-score–normalized radiomic features across all 61 patients, stratified by radiomic cluster (bottom annotation) and tumor subregion label (top annotation). Cluster 4 exhibits globally elevated radiomic intensity, especially in the edema and core regions. **(B–D)** Representative features enriched in Cluster 4 from different tumor compartments, box plots show z-scores across clusters; test = one-way ANOVA with Tukey *post-hoc*: **(B)** Shape sphericity (NET), **(C)** Run percentage (ED), **(D)** Dependence non-uniformity normalized (NET). Box plots show Z-score distributions across clusters. **(E)** Volcano plot comparing Cluster 4 to other clusters reveals 13 significantly enriched radiomic features, including texture- and volume-based metrics such as zone variance, mesh volume, and several small-area emphasis features. Volcano plot contrasts Group 4 (n = 6) *vs* others (n = 55); significance defined by FDR (Benjamini–Hochberg) on feature-wise tests. **(F)** Kaplan–Meier plots showing overall survival stratified by high *vs*. low values of three representative radiomic features. None demonstrated significant prognostic value individually, highlighting the value of composite radiomic subtypes.

To further characterize the biological relevance of the radiomic groups, we systematically examined the distribution of imaging features across anatomically defined tumor subregions. A heatmap of standardized radiomic profiles across all 61 patients demonstrated marked inter-group differences in feature intensity across these compartments, with the top annotation bar indicating radiomic group membership and the second annotation bar denoting tumor subregion labels (**Figure 2A**, **Supplementary Table 1**).

Group 4, previously identified as a high-risk group, displayed consistently elevated values for a broad set of radiomic features, particularly those derived from the core and peritumoral edema. These findings suggest that Group 4 tumors are characterized by more heterogeneous internal architecture and altered microenvironmental signatures at the imaging level. Among the features most enriched in Group 4, three were identified as representative markers: shape sphericity in the NET (**Figure 2B**), gray-level run percentage in the ED zone (**Figure 2C**), and dependence non-uniformity normalized in the ET (**Figure 2D**). Specifically, increased shape sphericity indicates a more rounded and geometrically uniform tumor core, which may reflect expansion in a confined anatomical space, possibly associated with aggressive but symmetric growth patterns. Elevated run percentage in the edema zone reflects a high degree of local texture homogeneity, implying that edema in Group 4 is structurally more organized or spatially constrained. Dependence non-uniformity normalized, a texture feature quantifying variability in the spatial dependence of pixel intensities, was significantly increased in the ET, indicating greater intra-regional heterogeneity within the actively proliferating component of the tumor.

Notably, none of the radiomic features extracted from the necrotic compartment showed statistically significant differences across groups, suggesting that necrosis contributes minimally to the stratification observed in the unsupervised analysis. A comprehensive volcano plot comparing Group 4 to all other groups identified 13 significantly enriched features in Group 4, including zone variance, gray-level non-uniformity, mesh volume, and several small-area emphasis metrics. These features collectively reflect increased morphological irregularity, textural heterogeneity, and complex spatial organization in Group 4 tumors, suggesting that Group 4 tumors exhibit radiomic patterns commonly associated with structural complexity, which may correlate with more aggressive biological behavior to be validated at the transcriptomic level.

However, despite the marked differences in radiomic profiles, none of these features individually exhibited significant prognostic value in univariate survival analysis. Kaplan–Meier curves for six top-ranked features demonstrated no statistically significant differences in overall survival between high- and low-value groups (**Figure 2F**), indicating that these radiomic variables are insufficient as standalone biomarkers for survival prediction.

This discrepancy highlights a critical limitation of purely image-based phenotyping: although radiomic groups may reflect integrated tumor states with prognostic significance, individual features fail to capture the underlying biological drivers of patient outcome. To resolve this gap, we next investigated whether the imaging-defined high-risk subtype corresponds to distinct transcriptional programs within the tumor and its microenvironment, through integrative analysis of matched bulk (same 61 patients) and single-cell transcriptomic data.

### 3.3 Transcriptomic profiling reveals a highly proliferative, mitosis-enriched gene signature in Group 4 tumors

**Figure 3:**
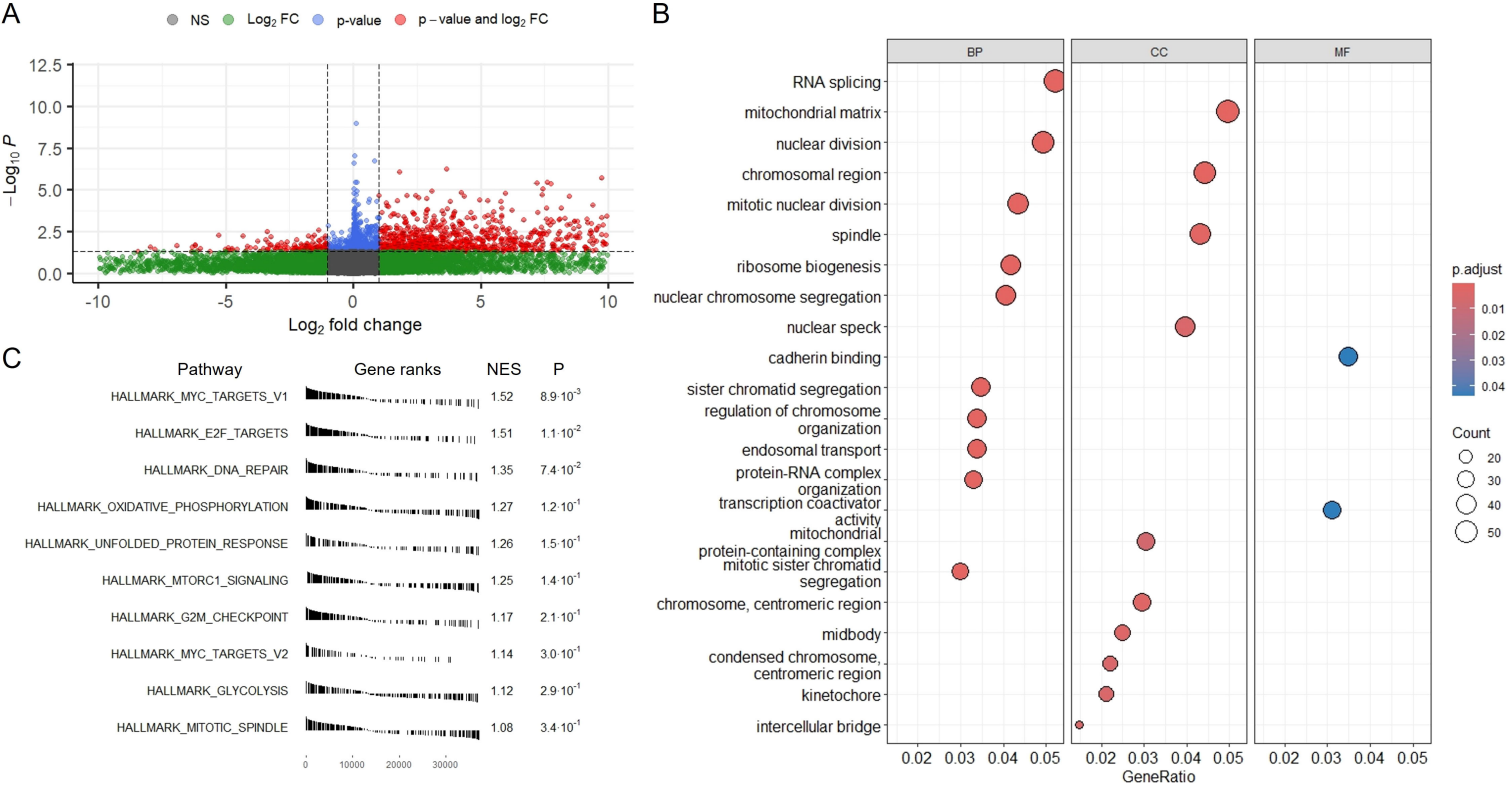
Transcriptomic profiling of radiomic Group 4 tumors reveals enrichment of proliferative gene programs. **(A)** Volcano plot of DEGs comparing Group 4 tumors to all other radiomic groups. Red dots indicate significantly upregulated genes, blue dots indicate significantly downregulated genes, and gray/green represent non-significant genes. Bulk RNA-seq matched to the same 61 MRI patients; differential expression by limma with Benjamini–Hochberg FDR control; DEGs defined as |log_2_FC| ≥ 1 and FDR < 0.05 (two-sided). **(B)** GO enrichment analysis of Group 4 DEGs across BP, CC, and MF categories. Top enriched terms include mitotic nuclear division, ribosome biogenesis, and chromosomal segregation, indicating increased cell cycle and biosynthetic activity. GO enrichment on FDR-filtered DEGs; pathway p-values FDR-adjusted (BH). **(C)** GSEA using MSigDB Hallmark gene sets. Top enriched pathways include MYC targets, E2F targets, DNA repair, and oxidative phosphorylation, all consistent with a highly proliferative and metabolically active tumor state. GSEA (MSigDB Hallmark) using preranked statistics; enrichment q-values are FDR-adjusted (BH).

Given the pronounced radiomic heterogeneity observed in Group 4, we next sought to determine whether these imaging-defined subtypes are underpinned by distinct transcriptional programs. To this end, we performed differential expression analysis between Group 4 tumors and all other radiomic subtypes. A total of 1,010 genes were significantly upregulated and 158 genes were downregulated in Group 4, using a log_2_ fold change threshold of ±1 and FDR < 0.05 (Benjamini–Hochberg adjusted) (**Figure 3A**).

To functionally characterize these DEG), we performed Gene Ontology (GO) enrichment analysis across three categories: biological process (BP), cellular component (CC), and molecular function (MF). The top enriched terms included mitotic nuclear division, sister chromatid segregation, chromosome segregation, ribosome biogenesis, RNA splicing, DNA replication, and nuclear chromosome segregation (**Figure 3B**). These annotations strongly indicate that Group 4 tumors are transcriptionally defined by heightened proliferative capacity and activation of chromosome dynamics programs. Enrichment of genes involved in mitotic spindle organization and RNA processing further supports the notion that Group 4 represents a transcriptionally active and cell cycle–engaged tumor state.

To further corroborate these findings, we conducted GSEA using the MSigDB Hallmark gene sets. The top enriched pathways included MYC targets V1, E2F targets, DNA repair, oxidative phosphorylation, and unfolded protein response (**Figure 3C**). These pathways are canonically associated with high proliferation, biosynthetic activity, and mitochondrial metabolism. Although not all gene sets reached nominal statistical significance at *p* < 0.05, several demonstrated normalized enrichment scores (NES) exceeding 1.5, and trends were consistent across related pathways. Notably, the enrichment of G2/M checkpoint, mitotic spindle, and MTORC1 signaling pathways further supports the interpretation that Group 4 tumors exhibit a hyperproliferative phenotype with active mitotic machinery and growth signaling cascades.

Taken together, transcriptomic analysis of Group 4 tumors revealed upregulation of core cell cycle regulators, mitotic apparatus components, and DNA replication genes, consistent with a transcriptional program characteristic of highly proliferative tumor cells. Enrichment of MYC and E2F target gene sets, along with pathways involved in chromosomal segregation, ribosome biogenesis, and oxidative phosphorylation, further supports a state of elevated biosynthetic and mitotic activity. However, bulk RNA-seq data do not resolve the cellular origin of these signals, making it unclear whether they primarily arise from malignant cells or from specific microenvironmental components. To address this limitation, we next leveraged single-cell RNA sequencing datasets to determine the cellular contributors to the proliferation-associated gene programs in Group 4 and to identify specific tumor or stromal subpopulations that may underlie its distinct radiogenomic profile.

### 3.4 Single-cell transcriptomic analysis links Group 4 programs to proliferative neural stem-like populations with pseudotemporal lineage progression

To investigate the cellular basis of the transcriptional programs enriched in Group 4, we analyzed publicly available single-cell RNA sequencing data from glioblastoma. This analysis aimed to identify the cell types most closely associated with Group 4 specific gene expression patterns and to evaluate whether these cell populations exhibit developmental trajectories that may account for the observed transcriptomic and radiographic heterogeneity.

**Figure 4:**
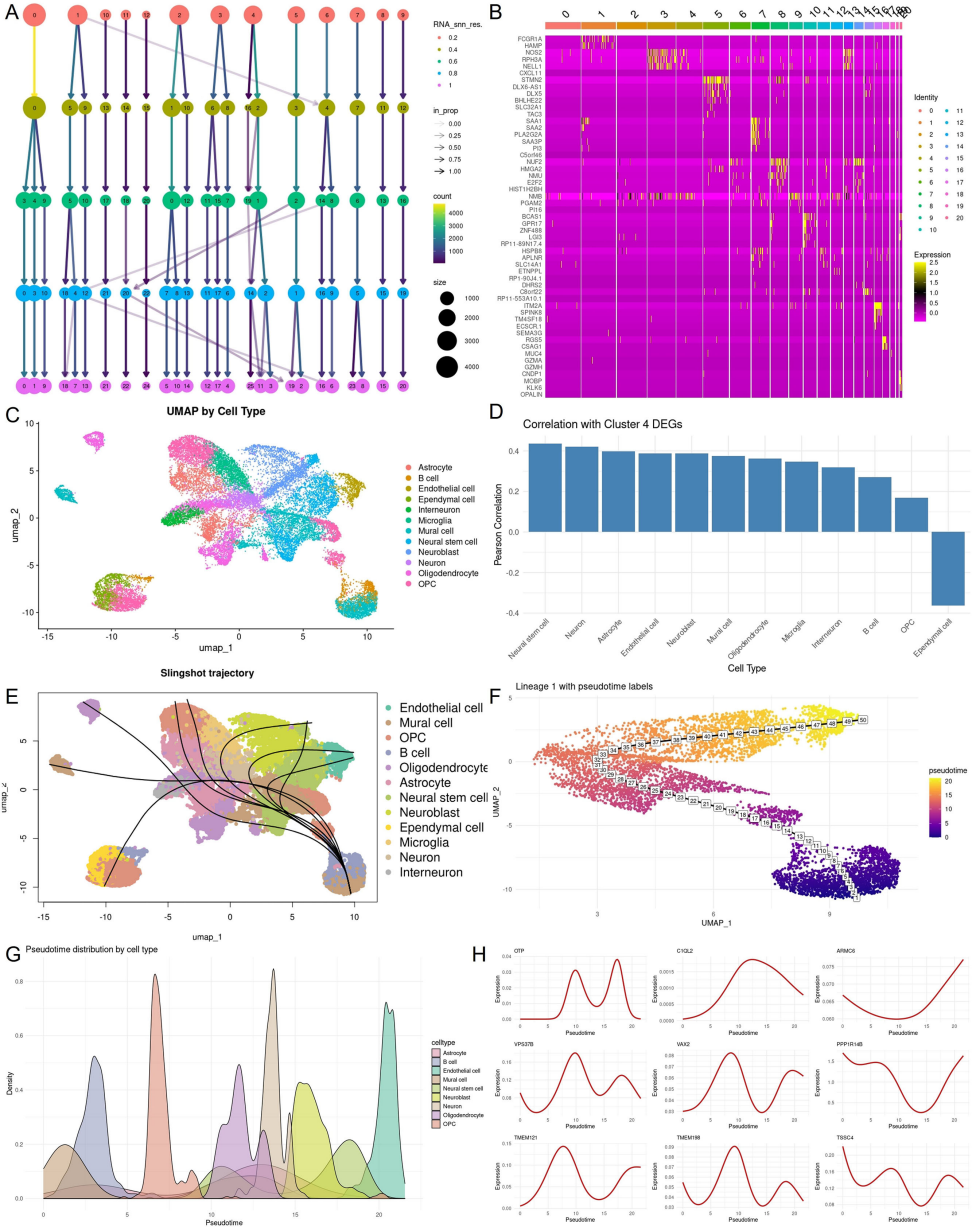
ScRNA-seq analysis reveals that Group 4 gene signatures are enriched in NSC–like populations. **(A)** Clustree plot displaying clustering resolution optimization. Resolution 0.6 was selected to define 22 transcriptionally distinct cell populations. **(B)** Heatmap showing canonical marker gene expression across identified clusters, enabling annotation of major glioblastoma-associated cell types. **(C)** UMAP plot of all single cells colored by annotated cell types, including neural stem cells, astrocytes, oligodendrocytes, neurons, and immune cells. **(D)** Pearson correlation coefficients between cell-type marker gene expression and Group 4 DEGs, highlighting NSC–like and neuroblast populations as most correlated. **(E)** Slingshot pseudotime trajectory analysis, with inferred lineage paths showing developmental progression from stem-like to differentiated cell states. **(F)** Visualization of lineage 1 with cells colored by pseudotime values, illustrating dynamic transitions among neural populations. **(G)** Distribution of pseudotime across annotated cell types, with stem-like and progenitor cells enriched at early pseudotime stages. **(H)** Pseudotime expression dynamics of selected Group 4 DEGs (e.g., VAX2, CDCA2, TPX2), demonstrating coordinated temporal activation patterns during lineage progression.

We first performed unsupervised clustering of single cells and assessed resolution stability using Clustree. A resolution of 0.6 was selected as optimal, yielding 22 transcriptionally distinct clusters (**Figure 4A**). The top three marker genes for each cluster are shown in **Figure 4B**. Cell types were annotated using canonical markers and scMayoMap reference mapping, identifying 14 major lineages including neural stem cells, neuroblasts, neurons, oligodendrocyte precursor cells, and various glial, vascular, and immune cell types (**Figure 4C**).

To assess which of these cell populations were most relevant to the transcriptional programs enriched in Group 4 tumors, we correlated the Group 4 specific DEG signature with marker gene expression across annotated cell types. Neural stem cells exhibited the strongest positive correlation, followed by neuroblasts and neurons, while endothelial and immune lineages showed minimal or negative correlation (**Figure 4D**). These results suggest that the cell types most associated with Group 4 specific gene expression, particularly neural stem cells and neuroblasts, represent undifferentiated neuroectodermal populations. Given that Group 4 DEGs are enriched for mitotic and biosynthetic processes (**Figure 3**), these findings implicate neural stem–like cells as a likely source of these transcriptional programs.

To further examine whether Group 4 associated cell types exhibit developmental dynamics that could underlie the observed transcriptional and radiographic heterogeneity, we performed pseudotime analysis centered on neural stem cells. These cells were positioned at the root of inferred lineage trajectories (**Figure 4E**), supporting their selection as the origin for trajectory inference. Lineage 1 was selected for downstream analysis due to its continuity from neural stem cells toward neuroblasts and neurons (**Figure 4F**).

The pseudotemporal structure was further supported by the distribution of annotated cell types across pseudotime, where neural stem cells were enriched at early stages and more differentiated neuronal populations appeared at later stages (**Figure 4G**). Several representative Group 4 DEGs demonstrated pseudotime-associated expression patterns. For example, OTP, C1QL2, and VAX2 showed early or peak expression during the transition from neural stem cells to neuroblasts. These genes have been implicated in neurodevelopmental regulation, including regional brain patterning and lineage specification, suggesting that Group 4 transcriptional signatures are enriched in undifferentiated, proliferative, and developmentally plastic cellular states (**Figure 4H**).

### 3.5 Spatially confined NSC–like niches define Group 4 tumor architecture

Given the strong association between Group 4 transcriptional programs and undifferentiated neuroectodermal lineages identified in single-cell analysis, we next asked whether these gene expression patterns exhibit spatially localized enrichment within glioblastoma tissue. Specifically, we investigated whether NSC–associated programs, implicated in Group 4, are confined to distinct tumor regions.

**Figure 5:**
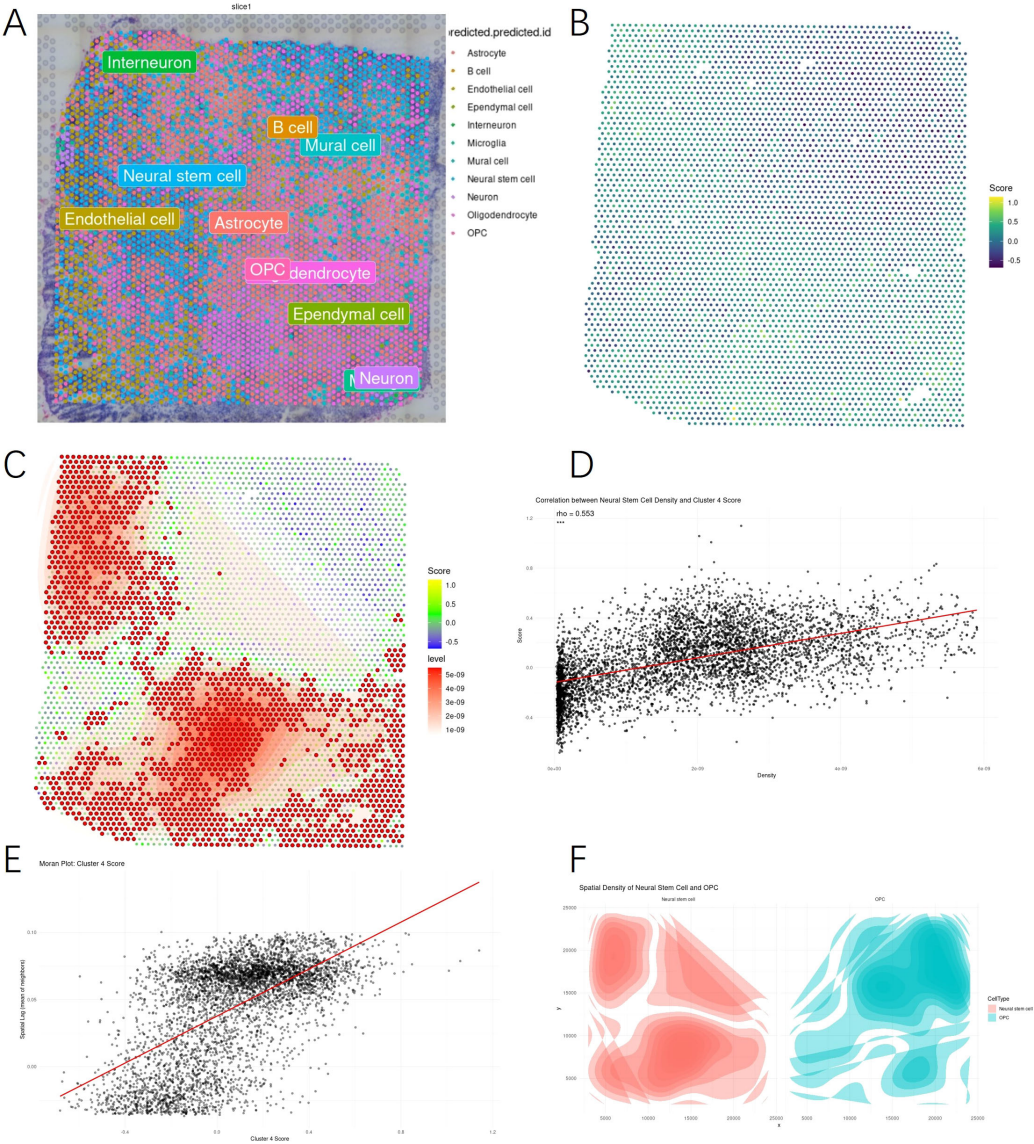
Spatial transcriptomics reveals enrichment of NSC–like populations in regions with high Group 4 gene expression. **(A)** Representative spatial transcriptomic map of a glioblastoma section, annotated with predicted cell types from deconvolution analysis. **(B)** Spatial distribution of Group 4 DEG module scores across the tissue section, with high scores localized to peripheral regions. **(C)** Hotspot analysis identifying significant spatial enrichment of Group 4 module scores using Getis-Ord Gi* statistics. **(D)** Correlation analysis showing a positive relationship between neural stem cell density and Group 4 module scores (ρ = 0.53). **(E)** Moran’s I spatial autocorrelation analysis demonstrating significant clustering of Group 4 module scores. **(F)** Density plots of NSC and OPC localization, indicating overlapping but distinct spatial niches.

To address this, we first applied deconvolution analysis to spatial transcriptomic data to infer the distribution of major cell types. The resulting cell type map revealed regional enrichment of NSCs and related neuroectodermal populations, such as interneurons and OPCs (**Figure 5A**). We then calculated a module score using Group 4 DEGs and visualized its spatial distribution (**Figure 5B**). Regions with high scores were non-uniformly distributed, suggesting potential spatial patterning.

Overlaying NSC density revealed substantial co-localization with Group 4 module scores, as highlighted by hotspot enrichment (**Figure 5C**). This spatial concordance was quantitatively supported by a positive correlation between NSC density and Group 4 score across spatial spots (ρ = 0.553, **Figure 5D**), and further confirmed by spatial autocorrelation analysis indicating non-random clustering (**Figure 5E**).

To examine potential niche exclusivity, we compared NSC and OPC spatial densities, which showed largely complementary patterns, suggesting that NSC-enriched regions are distinct from those dominated by OPCs (**Figure 5F**).

Notably, these NSC-like gene expression domains were primarily localized to the tumor periphery, a spatial pattern that aligns with imaging features of peripheral enhancement and suggests a potential role in driving boundary expansion or invasion. This spatial confinement to the tumor edge highlights a proliferative and undifferentiated niche that may contribute to therapeutic resistance and recurrence, underscoring its significance as a biologically and clinically relevant compartment within Group 4 tumors. This correspondence was evaluated at a macroscopic level, comparing regional enrichment patterns rather than direct spatial registration, as the physical resolution of spatial transcriptomics (tens of microns) is substantially higher than that of MRI (millimeter scale). Thus, the observed alignment represents a multi-scale association between molecular niches and imaging-defined tumor architecture.

### 3.6 VAX2 knockdown inhibits glioma cell proliferation

**Figure 6:**
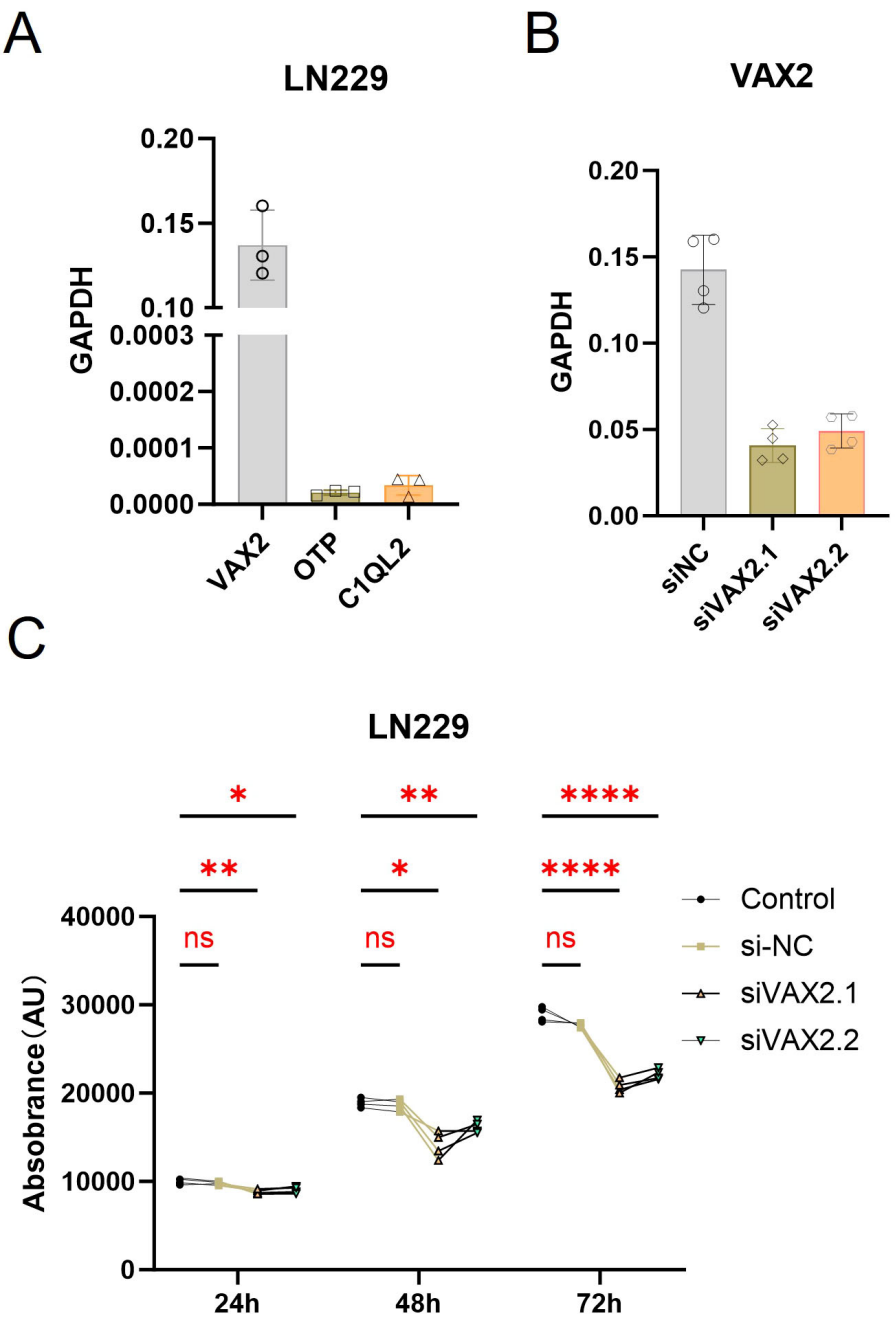
Functional validation of VAX2 in glioblastoma cell proliferation. **(A)** qRT-PCR analysis of VAX2, OTP, and C1QL2 expression in LN229 cells. VAX2 showed robust expression, whereas OTP and C1QL2 were expressed at levels indistinguishable from background, and were excluded from further functional validation. **(B)** Validation of siRNA-mediated knockdown efficiency of VAX2 at 48 hours post-transfection. Both siVAX2.1 and siVAX2.2 significantly reduced VAX2 mRNA levels compared to siNC. **(C)** Cell viability assay (CCK-8) performed at 24, 48, and 72 hours post-transfection. Knockdown of VAX2 led to a significant reduction in cell proliferation compared to siNC and untreated control groups at all time points. Data represent mean ± SD of four independent replicates. Statistical significance was determined by one-way ANOVA with Tukey’s *post hoc* test (*p < 0.05; **p < 0.01; ****p < 0.0001; ns, not significant).

To investigate the biological relevance of candidate transcription factors enriched in the NSC-like compartment, we next performed functional validation of OTP, C1QL2, and VAX2 using the LN229 glioblastoma cell line (ATCC, CRL-2611). Quantitative RT-PCR confirmed that both OTP and C1QL2 were expressed at very low levels in LN229 cells (**Figure 6A**), with Ct values approaching those of RT– controls. This low expression may reflect cell line limitations or context-specific expression restricted to *in vivo* settings, such as patient-derived or spatially distinct tumor regions. Due to this ambiguity, we excluded OTP and C1QL2 from further *in vitro* analyses and focused subsequent experiments on VAX2, which showed robust expression in LN229.

To assess the knockdown efficiency, we transfected LN229 cells with two independent siRNAs targeting VAX2 and measured mRNA levels 48 hours post-transfection. Both siVAX2.1 and siVAX2.2 significantly reduced VAX2 transcript abundance compared to siNC (**Figure 6B**), validating their efficacy.

We then evaluated the impact of VAX2 knockdown on LN229 cell proliferation using an ATP-based assay at 24, 48, and 72 hours post-transfection. VAX2 silencing led to a consistent reduction in cell growth at each time point, with statistically significant differences observed by 48 and 72 hours (**Figure 6C**). These results suggest that VAX2 plays a functional role in promoting glioma cell proliferation and may contribute to the proliferative potential of NSC-like tumor subpopulations in Group 4.

## 4 Discussion

Our integrative analysis of radiologic and transcriptomic data provides new evidence linking imaging heterogeneity to distinct molecular programs in GBM. We found that radiomics-based clustering of preoperative MRIs can stratify patients by outcome, identifying a particularly aggressive subgroup (Group 4) characterized by a peripheral rim-enhancing radiographic phenotype and markedly poorer survival. Molecularly, this high-risk imaging phenotype corresponded to transcriptional programs enriched for cell cycle regulators and NSC–like signatures, indicative of a highly proliferative, undifferentiated cell state. Using single-cell and spatial transcriptomics, we further showed that these proliferative stem-like tumor cells are not uniformly distributed but instead concentrate at the tumor’s invasive edges. This spatial pattern aligns closely with the MRI features of Group 4 tumors, providing a biological basis for the imaging-defined risk subtype. These findings build on foundational radiomics/radiogenomics work establishing that quantitative imaging patterns can reflect tumor biology and prognosis in GBM and other cancers (24, 25). Our study extends this framework by linking a preoperative MRI phenotype to paired transcriptomic programs at the patient level.

Our spatial transcriptomic data illustrates that the high-risk radiomic signature (Group 4) and its associated stem-like gene program are concentrated at the tumor periphery. Single-cell and spatial datasets are used here as hypothesis-generating context rather than cohort-level confirmation. In the representative tumor shown, the radiomic risk score is elevated in peripheral regions (green/yellow points indicate a high Group 4 score), co-localizing with areas of elevated neural stem cell–like gene expression (red shading) at the invasive edge. By contrast, the tumor core shows low radiomic risk scores and minimal stem-like transcriptional signals (blue areas). This concordance between the imaging phenotype and molecular profile supports the notion that rim-enhancing regions on MRI correspond to biologically distinct, stem cell–rich tumor habitats at the margin.

The enrichment of a NSC–like, highly proliferative cell population in peripheral tumor regions offers a compelling explanation for the worse outcomes observed in Group 4 patients. Glioma stem-like cells have long been implicated in driving GBM aggressiveness and recurrence (26), and these cells tend to inhabit niches at the tumor margin and invasive fronts. Our findings are in line with this paradigm: the radiographically evident rim of enhancement in Group 4 tumors corresponds to a tumor compartment dominated by invasive, stem-like cells at its periphery. Consistently, other investigators using high-resolution spatial profiling have reported that GBM cells infiltrating into adjacent brain tissue upregulate neurodevelopmental pathways and glial lineage programs (4). This convergence of evidence suggests that advanced imaging can map the distribution of clinically important cell states within a tumor noninvasively. In this case, it can identify tumors that harbor aggressive, stem-like cells at their edges. This radiogenomic correspondence aligns with prior reports that radiographic patterns can map clinically relevant cell states and microenvironmental niches in GBM (27).

Notably, our radiomics-driven stratification appears to capture an axis of tumor biology that cuts across the traditional bulk transcriptomic subtypes of GBM. The classical, mesenchymal, and proneural transcriptional subtypes defined in prior studies are each composed of mixtures of malignant cell states, including a neural progenitor–like (NPC-like) state (28). In our cohort, Group 4 tumors likely spanned multiple of these conventional subtypes, yet convergently exhibited a predominance of the NPC-like, stem-associated program. This highlights how an imaging-based classification can reveal biologically meaningful distinctions that might be obscured when analyzing whole-tumor averages. Our imaging–molecular links are based on patient-matched bulk RNA-seq with Benjamini–Hochberg FDR control, thereby reducing the risk of spurious associations. Embracing phenotypic classification approaches has been proposed as a way to better represent GBM’s heterogeneity (29), and our results affirm that an imaging phenotype can serve as a surrogate for an aggressive molecular profile. In essence, radiomics provided a different lens on tumor classification, one that identified a high-risk state defined by cell-intrinsic properties (stemness and proliferation) and spatial context (tumor edge) rather than by traditional histopathological features alone.

One molecular finding in our high-risk subgroup was the transcription factor VAX2. VAX2 is a homeobox gene involved in neural development, and it has not been widely studied in glioblastoma. Interestingly, recent work in other cancers suggests an oncogenic role for VAX2, for example, VAX2 was found to be significantly upregulated in gastric cancer and to promote tumor cell proliferation and invasion (30). In our analysis, VAX2 expression was elevated in Group 4 tumors, and we hypothesized that it might drive the proliferative, stem-like phenotype of this subtype. To test this, we inhibited VAX2 in GBM cells *in vitro*. Functional assays were limited to LN229 *in vitro*. *In vivo* studies were not performed because animal ethics approval could not be obtained within the study period; future validation in multiple GBM models and xenografts is warranted.

LN229 glioblastoma cells transfected with two independent VAX2-targeting exhibited significantly lower viability over 72 hours compared to cells transfected with a siNC. In the growth curves shown, VAX2 knockdown cells grew more slowly, with a ~30–40% reduction in cell proliferation by 72 h (*p* < 0.01), indicating that suppression of VAX2 inhibits GBM cell growth *in vitro*. This result confirms that VAX2 enhances the proliferative capacity of GBM cells, supporting the notion that VAX2 contributes to the aggressive biology of the Group 4 subtype.

From a clinical perspective, our findings highlight the potential of advanced MRI analytics to improve GBM patient stratification and treatment planning. Incorporating radiomic analysis into the pre-surgical workflow could enable identification of patients with a high-risk imaging phenotype (such as Group 4) at diagnosis, who might benefit from intensified therapy or enrollment in clinical trials. Indeed, previous studies have demonstrated that radiomics-based models can successfully stratify GBM patients by survival risk in independent cohorts (31). Moreover, the tight spatial correspondence between imaging features and tumor biology suggests that radiomics might help guide more tailored interventions. For example, if a tumor displays the peripheral enhancement pattern characteristic of Group 4, clinicians might consider extending the surgical resection margin or delivering boosted radiation to the tumor rim, given that our data indicate an abundance of aggressive stem-like cells in that region. Targeting these residual peripheral cells is critical, as they are likely drivers of post-surgical recurrence (32–34). In fact, recent studies have emphasized the importance of therapies directed at the invasive margins of GBMs (4), where therapy-resistant, migratory tumor cells reside. Additionally, the discovery of VAX2 as a potential promoter of proliferation opens a new avenue for precision oncology: while directly targeting a transcription factor like VAX2 is challenging, its downstream effectors or regulatory network could be investigated for druggable targets, and VAX2 expression itself may serve as a biomarker for identifying patients with particularly aggressive, stem-cell-rich tumors.

This study has several limitations. First, although survival models adjusted for age and sex, additional clinical covariates (e.g., extent of resection, MGMT status, and treatment regimen) were unavailable in TCIA, and residual confounding cannot be excluded. Second, manual segmentations were adjudicated by a senior neurosurgeon and used as consensus masks, but formal Dice or ICC values were not computed. Third, feature harmonization with ComBat was not applied due to incomplete acquisition metadata. Fourth, the sample size (n = 61) was modest; however, internal stability analyses using bootstrap resampling and consensus clustering demonstrated high within-cluster co-assignment probabilities (mean > 0.8), supporting the robustness of the four-group structure. Fifth, external validation in an independent imaging cohort with matched transcriptomic and clinical data was not feasible because such datasets remain scarce. We are currently assembling a multi-institutional dataset to test whether the Group 4–like imaging phenotype and its associated transcriptomic program can be reproduced in independent cohorts. Sixth, functional validation of VAX2 was restricted to a single GBM cell line with no *in vivo* experiments because animal ethics approval was not available during the study period. Finally, while single-cell and spatial data support biological plausibility, they were used as hypothesis-generating context rather than for cohort-level confirmation.

Looking ahead, our study suggests several future directions. Prospective validation in larger, independent GBM cohorts is needed to confirm the robustness of the radiomics-defined subtypes and their transcriptomic signatures. Technical refinements such as harmonizing MRI acquisition, improving segmentation (potentially with machine learning), and applying reproducibility indices will be critical for standardization. Mechanistic studies, including single-cell lineage tracing and *in vivo* models, should clarify how the stem-like, VAX2-positive population contributes to therapy resistance and recurrence, as well as its interactions with the immune microenvironment. Finally, these insights could guide therapeutic development: patients with the high-risk peripheral enhancement phenotype may be candidates for intensified local therapy, while VAX2-regulated pathways may inform novel targeted strategies. Integrating radiomic classifiers into preoperative workflows could ultimately help personalize treatment planning.

In conclusion, this study demonstrates the value of combining radiomics with multi-dimensional transcriptomics to elucidate the biological underpinnings of GBM imaging phenotypes. By revealing a spatially localized, stem-like tumor cell program associated with an adverse radiographic subtype, our work suggests that noninvasive imaging can stratify patients by tumor biology and potentially guide more informed, personalized therapeutic strategies.

## Data Availability

The datasets analyzed in this study are publicly available. Radiological and clinical data for glioblastoma patients were obtained from The Cancer Imaging Archive (TCIA, https://www.cancerimagingarchive.net/) and The Cancer Genome Atlas (TCGA-GBM, https://portal.gdc.cancer.gov/). Single-cell and spatial transcriptomic datasets were retrieved from the Gene Expression Omnibus under accession numbers GSE103224 and GSE194329.

## Supplementary material

Supplementary Table 1 Standardized radiomic profiles.

Supplementary Data Sheet 1 Radiomics Workflow Description.
