# Supplementary material for: MRI-based radiomic clustering identifies a glioblastoma subtype enriched for neural stemness and proliferative programs
Source: Front Oncol. 2025 Nov 25;15:1662401. doi: 10.3389/fonc.2025.1662401 (PMC12685660; doi:10.3389/fonc.2025.1662401)
Supplement: Supplementary Data Sheet 1 — Radiomics Workflow Description. [file DataSheet2.pdf]

Radiomics Workflow Description

Image Acquisition and Preprocessing

Source: Pre-operative T1-weighted post-contrast (T1CE) MR images from TCGA-GBM (via TCIA)

Format: DICOM converted to NIfTI (.nii.gz)

Preprocessing:

Resampled to 1×1×1 mm³ isotropic voxel size

Intensity normalization using Z-score (within brain mask)

Bias field correction via N4ITK (optional)

Skull stripping not applied (masks were manually verified)

```
import SimpleITK as sitk

def resample_image(image, new_spacing=[1,1,1]):
    original_spacing = image.GetSpacing()
    original_size = image.GetSize()
    new_size = [
        int(round(osz*ospc/nspc)) for osz, ospc, nspc in zip(original_size, original_spacing, new_spacing)
    ]
    resampler = sitk.ResampleImageFilter()
    resampler.SetOutputSpacing(new_spacing)
    resampler.SetSize(new_size)
    resampler.SetInterpolator(sitk.sitkLinear)
    return resampler.Execute(image)
```

Segmentation and Regions of Interest (ROI)

Segmentation: Manual or semi-automated delineation using 3D Slicer

Regions:

Enhancing tumor (ET)

Non-enhancing core (NET)

Peritumoral edema (ED)

Saved as individual .nii.gz label masks (one per region).

Feature Extraction

Tool: PyRadiomics

Feature Classes:

First-order statistics

Shape (3D)

GLCM, GLRLM, GLSZM, NGTDM, GLDM

Image Filters Applied: Original, Wavelet, LoG (sigma = 3.0)

```
from radiomics import featureextractor

extractor = featureextractor.RadiomicsFeatureExtractor()

extractor.enableAllImageTypes()

extractor.enableAllFeatures()

result = extractor.execute("T1CE_resampled.nii.gz", "tumor_mask.nii.gz")
```

Total features per region: ~1,200

Missing values: Features with NaNs removed before analysis

Feature Selection and Clustering

Pre-selection:

Variance thresholding (threshold=0.01)

Highly correlated features removed (|r| > 0.9)

Normalization: Min-Max scaling to [0,1]

Clustering: PCA → K-means (k=4)

Stability assessment: Consensus clustering (100–150 bootstraps; 90% samples × 80% features per iteration)

Reproducibility & Inter-reader Consistency

Inter-reader reproducibility: Not directly tested in this study; future validation planned

Repeatability assumption: All images and masks derived from standardized TCIA dataset with expert segmentation

Tool Versions:

Python: 3.9

PyRadiomics: 3.0.1

scikit-learn: 1.3.0
